# Test-Retest Reliability of Measurements of the Center of Pressure Displacement in Quiet Standing and During Maximal Voluntary Body Leaning Among Healthy Elderly Men

**DOI:** 10.2478/v10078-011-0018-9

**Published:** 2011-07-04

**Authors:** Stemplewski Rafał, Maciaszek Janusz, Osiński Wiesław, Szeklicki Robert

**Affiliations:** 1 Department of Theory of Physical Education and Anthropomotoric, University School of Physical Education in Poznań, Poland

**Keywords:** aging, center of pressure, limits of stability, reliability

## Abstract

The aim of the study was to evaluate intra- and intersession test-retest reliability for the measurements of centre of pressure displacement in quiet standing and during maximal voluntary body leaning (approximate area of stability limits).

27 elderly men participated in the study (71.4±4.9 of age). Intrasession (4 measurements with two-minute breaks) and intersession (4 measurements one week apart) reliability were examined. Parameters connected to the centre of pressure data (AMTI force platform) were measured during a quiet stance and voluntary body leaning in medio-lateral and anterior-posterior directions (approximate limits of stability). Intraclass correlation coefficients (ICC_2,1_ and ICC_2,k_) were calculated.

In case of quiet standing, only mean velocity of centre of pressure sway provides high reliability in intrasession – ICC_2,1_ of .84 and ICC_2,k_ of .96, and in intersession – ICC_2,1_ of .76. Evaluation of limits of stability showed high values of all parameters (maximal and minimal displacement in sagittal and frontal planes, distance between maximal and minimal position of centre of pressure in sagittal and frontal planes and approximated area of stability limits) in intrasession – ICC_2,1_ between .82 to .96 and ICC_2,k_ between .95 to .99. Similar tendency was observed in the intersession retest.

Average velocity of the centre of pressure is the only parameter that showed a high application value in case of measurements during quiet standing. Parameters related to the stability limits appeared very reliable what proves that this evaluation may have potential application in the clinical practice.

## Introduction

Studies regarding body balance assessments are often focused on finding methods which would allow to evaluate the risk of falls among older people. Practical tests which did not require the implementation of research equipment, such as Performance Oriented Assessment of Mobility Problems (Tinetti, 1986), and Clinical Test for the Sensory Interaction on Balance (Shumway-Cook & Horak, 1986) were conducted. Additionally, posturographic methods based on the measurement of the displacement of the centre of pressure (COP) have been developed. The application of force platforms provides us, as a result, with comprehensive data related to the oscillation of the COP. The reliability of such studies is however limited by the reference to evaluation of the risk of falls of older people. Generally, we focused on studies conducted in quiet stance positions (static conditions) and voluntary performance of movements (dynamic/functional conditions) in posturographic studies. Additionally, in the case of a quiet stance, interference of individual signal systems is implemented, including for instance closed eyes, or standing on unstable foam surface (Kim et al., 2008). The comparison of data reveals some discrepancies which result in the fact of using platforms of varied construction and various softwares of different parameters. It is pointed out that, apart from average displacement and amplitude of the COP, parameters concerning the average velocity/total path length of COP (and their derivatives in anterior-posterior - AP and medio-lateral - ML directions) may constitute an informative value within the scope of relations between posturographic characteristics and the risk of falling down (Piirtola & Era, 2006). Moreover, these characteristics display the highest, among all stabilographic parameters, reliability rates in test-retest studies in older subjects (Lafond et al., 2004). On the other hand, however, higher results within the range of parameters of COP displacement do not have to be related with the reduction of postural stability (Corriveau et al., 2001). More precise information is provided by a simultaneous analysis of COP signals and the centre of mass (COM), as well as the evaluation of differences between them (Winter et al., 1998). It is reported that useful data concerning postural control may be obtained by analyzing the amplitude of the lengths of COPCOM displacement (Corriveau et al., 2001) or the ratio of the lengths COP/COM displacement (Błaszczyk, 2008).

As a consequence of a theoretical assumption regarding the posture stability model which compares a human being to an inverted pendulum, a need of the assumption concerning so called limits of stability (LOS) arises. The LOS mark the area in which a vertical projection of the COM may relocate without losing the balance of the body (Nashner & Mc Collum, 1985). In other words, the limits of stability constitute a potential scope of angular sway of the body from the perpendicular (Geldhof et al., 2006). According to the inverted pendulum theory presenting human body stability explanation (Gurfinkel, 1973; Winter et al., 1988), the bigger scope of potential sway is, the better stability becomes and the lower is the risk of fall.

In the evaluation of the reliability of the LOS study, most commonly, different variations of visual feedback were applied. The subjects were supposed to translocate the visualization of COP (in the form of a moving point) to strictly defined target positions. The studies focused on evaluation of accuracy of performing the task which was measured, among others, as reaching the destination, the time required to reach individual points and the consistency between the translocation and the defined direction (Brouwer et al., 1998; Geldhof et al., 2006; Maciaszek et al., 2007). Correspondingly to studies concerning postural sway, the usage of various research tools constituted a problem, which referred in particular to different protocols of research. From the perspective of the evaluation of the risk of falls, research protocols based on the definition of LOS seem to be very attractive, however, the selection of the appropriate procedure remains still an open question. Such research shall provide evaluation of the scope of the risk of falls simultaneously fulfilling the criteria of accuracy and reliability. At the same time, if such research is to offer a practical aspect, it seems that the procedure of its conducting, as well as interpretation of its results, shall be practicable (for both the subjects and the investigators). Former studies concentrated on differences in postural stability falling and non-falling elderly men (Stemplewski et al., 2003). As the indicator, a simplified version of LOS study relation to displacement of COP was adopted (LOS_COP_). However, feedback was not provided and the COM displacement was not evaluated. The studies revealed that fallers obtained statistically lower results than non-fallers in that parameter. The parameter may be considered as an indicator of a potential risk of falls.

The aim of the study was to evaluate intra- and intersession test-retest reliability for the measurement of COP displacement in quiet standing and during maximal voluntary body leaning (approximate area of stability limits).

## Material & methods

### Participants

27 elderly men participated in the study (71.4±4.9 of age, the range of 65–81). The subjects of the study were residents of Poznań, Poland. The lowest age limit classifying subjects to be included in the study was 65, that is the initial stage of the distinction in the age category “young-old” according to Spirduso (1995). The participation in the study was held on voluntary basis. Purposes, as well as a detailed schedule of the study, were presented during the recruitment process. Subjects who were interested in participation in the experiment signed their consent for taking part in the study.

The subjects of the study were selected out of 75 volunteers. The criteria of selection were as follows: independent functioning and in particular, independent moving, as well as no neurological, muscular or orthopedic deficits (according to the declarations of subjects).

Basic somatic features were measured. Body height, body mass and BMI equaled respectively 169.6±7.1 cm (157–187.3 cm), 71.4±11.9 kg (60–111 kg), 28.2±3.1 kg/m^2^ (23.3–38.4 kg/m^2^).

Local Ethics Committee issued its consent to conduct the research (resolution nr 770/06).

### Protocol

The study was divided into two parts. In the first part, COP displacement was measured in a quiet standing position (QS). In the second part, the evaluation of approximate value of LOS_COP_ (Stemplewski et al., 2003) during voluntary body leaning in sagittal and frontal planes was conducted (area calculated on the basis of length of maximal COP displacements forward, backward and sidewards). The research was carried out always by the same researcher in the same laboratory conditions. Each measurement lasted 30 seconds.

In case of QS and LOS_COP_, two variations of test-retest reliability evaluation were implemented. They were: a) intrasession reliability and b) intersession reliability.

- Intrasession reliability was conducted on the same day during one session – 4 trials (Santos et al., 2008). Each person was examined four times with two-minute breaks (sitting on a chair) between the repetitions. This type of evaluation of reliability is related to a random variability of the measurement per se. Thomas and Nelson (1990) claim that an immediate retest conducted during one session reflects the internal consistency, and its results referring to reliability are higher than in case of the intersession retest.
- Intersession reliability was conducted on the base of 4 trials one week apart, always at the same time of a day. Retest after longer periods depicts variability depending on implemented procedures and reflects the stability of the phenomena (Thomas & Nelson, 1990). The analysis of reliability focuses only on those parameters which were characterized by a high internal consistency (ICC≥ .75).

### Instrumentation

COP data were collected using the force plate (AMTI, AccuGait, USA). The sampling frequency amounted to 50 Hz. The platform was connected to the computer with the Balance Trainer software provided by the manufacturer.

#### Measurements

All the measurements were performed in standard conditions. Only the investigator and the subject were in the room during the measurement. The force platform was placed on a flat, stable surface. The subjects stepped onto the platform barefoot.

- In case QS, the subjects were to stand as still as possible for 30 seconds (Pinsault & Vuillerme, 2009; Salavati et al., 2009). They were instructed to stand quietly with upper limbs along the torso and look forward. The following parameters related to the displacements of COP were taken into consideration:
  - Y_Range_ – range in anterior-posterior (AP) direction (distance between maximal and minimal position of COP in sagittal plane),
  - X_Range_ – range in medio-lateral (ML) direction (distance between maximal and minimal position of COP in frontal plane),
  - MR – average radial position vector length (mean radius),
  - V_Avg_ – average velocity,
  - Area – sway area limited with an ellipse of the 95^th^ percentile.
- In case of evaluation of LOS_COP_, maximal voluntary forward, backward, leftward and rightward leaning of the whole body were measured. Leans were performed with the security against fall – the investigator stood next to the subject on the side of actual leaning and was ready to support the subject in case of loosing balance). The test started when the subject was standing in an upright position (for about 3s). Subjects were instructed to perform leaning slowly and attempt to keep the body extended in femoral joints and not to separate feet from the surface. At first, the instruction „please lean forward” was given, then the subject leaned maximally forward. Subsequently, the subject was requested to come back to the initial position (upright position). Consequently, an analogical procedure was implemented regarding backward, leftward and rightward leans. All four leans were performed during one trial. The following parameters related to the displacement of COP were taken into consideration:
  - F_max_ – maximal displacement from the centroid data in sagittal plane (forward),
  - B_max_ – minimal displacement from the centroid data in sagittal plane (backward),
  - FB – distance between maximal and minimal position of COP in sagittal plane,
  - R_max_ – maximal displacement from the centroid data in frontal plane (rightward),
  - L_max_ – minimal displacement from the centroid data in frontal plane (leftward),
  - RL – distance between maximal and minimal position of COP in frontal plane,
  - LOS_COP_ – deltoid area with diagonals of FB and RL [LOS_COP_=(FBxRL)/2]

### Statistical analysis

For the purpose of the intra- and intersession evaluation ICC_2,1_ coefficients were calculated (Shrout & Fleiss, 1979) on the basis of the two-way ANOVA. This formula allowed to compare results of within-subject and between-subject variability

$${\mathrm{ICC}}_{2,1}=\frac{{\mathrm{MS}}_{B}-{\mathrm{MS}}_{E}}{{\mathrm{MS}}_{B}+\left(n-1\right){\mathrm{MS}}_{E}+n\left({\mathrm{MS}}_{R}-{\mathrm{MS}}_{E}\right)/k},$$

where *MS_B_*, *MS_R_*, and *MS_E_* are mean squares of the two-way ANOVA, *n* represents the number of subjects and *k* - the number of tests.

Both results of single ratings (ICC_2,1_) and averages of *k* ratings (ICC_2,k_) in intrasession retest and results of single ratings in intersession retest were taken into consideration. In the case of single sessions, the value *k* of the number of tests, which had to be averaged to achieve ICC≥ .90, was calculated on the basis of rearranged Spearman-Brown formula:

$$k=\frac{R^{*}\left(1-R\right)}{R\left(1-R^{*}\right)},$$

where *R* is the received value, and R* is the target value of the reliability coefficient.

ICC analyses were computed by means of SPSS 18.0 software.

## Results

Table 1 presents results of ICC_2,1_, ICC_2,k_, and *k* for COP displacement measures obtained during the intersession and intersession retest.

Adopting the interpretation of the ICC value suggested by Fleiss (1986) – ICCs excellent > .75; ICCs fair to good > .40 and < .75; ICCs poor < .40 – it has been found that in the case of COP displacement during quiet standing, only V_Avg_ provides excellent reliability for single ratings – ICC of .84. Other parameters (maximal range in AP and ML directions, mean radius and sway area) revealed fair to good reliability – ICCs between .42 – .61. However, highest variability was found in the COP range in the AP direction (ICC of .42) and mean radius (ICC of .46). ICCs for averaged results of four tests were higher (between .74 to .96) with analogical tendency (highest values for V_Avg_ – ICC of .96 and lowest in case of Y_Range_ and MR – .74 and .77, respectively). The analysis of the *k* value of trials, necessary to be averaged in order to obtain ICC≥ .90, revealed that in case of V_Avg_, two trials were sufficient, whereas in case of other parameters, it was necessary to conduct between six and thirteen trials. V_Avg_ as the parameter of the highest reliability in intrasession retest was also evaluated with respect to the results stability in case of intersession retests (4 trials one week apart). An excellent ICC value of .76 was observed.

The analysis of results related to the evaluation of approximate values of LOS on the basis of displacement of COP during voluntary whole body leaning (Table 2) showed a very high level of internal consistency between all parameters evaluated in intrasession retests for single ratings (ICCs between .82 to .96), as well as for mean values of four trials (ICCs between .95 to .99). Additionally, it has been reported that for most parameters, the execution of one trial allows to obtain ICC ≥ .90. Only in case of maximal forward and maximal backward displacements of COP from the centroid data, it is necessary to average two trials.

In case of the evaluation of the stability of the results obtained in the intersession retest, excellent reliability for most examined variables was reported (ICCs between .78 to .85), with the exception of B_max_ (ICC of .67).

## Discussion

The purpose of the study was to provide evaluation of intra- and intersession test-retest reliability for the measurement of COP displacement in quiet standing and during maximal voluntary body leaning (approximate area of stability limits).

In case of QS, it has been found that only COP average velocity presents appropriate reliability in a single evaluation and averaging of two trials is sufficient to obtain ICC value ≥ .90 (X_Range_, Y_Range_, MR, and Area occurred not to be reliable). Average velocity showed also high reliability in intersession retests. Similar results were obtained by Lafond et al. (2004) who examined intrasession reliability of COP measures among elderly people at various time lengths of tests (30, 60 and 120 s). They reported that in every case ICC was higher for COP velocity both in AP and ML directions, whereas the reliability of the measurement increased as the time of the test was prolonged. On the other hand, the COP range in AP and ML directions as well as the sway area showed poor reliability. Lin et al. (2008) reported high ICCs for the mean velocity of COP in intrassesion and intersession test in elderly persons, too. However, they showed high ICC in case of sway area for intra- and intersession. Similar results were not obtained in own study, however, Lin et al. (2008) implemented in their study a different manner of evaluation of the sway area (area unit per time unit– mm^2^/s).

Thus the results showed that only average velocity of COP displacement may have a potential application value in evaluation of body balance among elderly when using a 30 s trial. High internal consistency and stability of results show that it can be used for actual status measures as well as evaluation of possible changes in time. The use of the other parameters (connected to spatial distribution of COP displacement) is questionable unless the results of greater number of trials (from 6 to 13) would be averaged. Although Lafond et al. (2004) showed the higher reliability of a 120 s trial, but standing still for prolonged time (as well as an increased number of trials) may be difficult for the elderly (e.g. possibility of attention distraction or movement) especially in case of examination of unhealthy or impaired people.

The second part of the study concerned the analysis of reliability of the COP parameters obtained during maximal voluntary leaning of the whole body in AP and ML directions, and particularly the calculated approximate area of stability limits (LOS_COP_). It has been reported that all the examined parameters showed a high level of internal consistency for single ratings and average ratings in intrasession retest. The analysis showed that only in case of forward and backward deflections, there was a need to conduct two trials to be averaged in order to obtain ICC ≥ .90. In other cases, a single trial allowed to conduct a sufficiently reliable measurement. The higher variability (but still in range of excellent reliability) within the range of F_max_ and B_max_ may be caused by the wider range of COP displacement in the AP direction. Analyzing distance between maximal and minimal position of COP in sagittal plane (FB) where the significance of the initial position and range of COP are eliminated, high results of ICC were noticed. A similar situation appeared in case of intersession tests, confirming high stability of obtained results.

Research focused on the evaluation of LOS is the consequence of the generally adopted model of stability – inverted pendulum – on the basis of which it is evident that the center of mass of the body may be „safely” displaced within strictly defined limits (Gurfinkel, 1973; Winter et al., 1988). In that context, the suggested method of LOS_COP_ measurement does not constitute a precise reflection of the definition of LOS as it does not refer to the evaluation of COM displacement. In spite of the fact that the research reveals high consistency between COP and COM signals (Benda et al., 1994), the situation concerns static condition – a quiet stance. In the case of displacement of the mass, the consistency significantly decreases, thus, one should be very cautious with calculations of the stability limits on the basis of the COP signal, which characterizes moments of forces implemented by the person examined (Juras et al., 2008). On the other hand, however, one should answer the question whether from the perspective of a clinical value (the evaluation of the risk of falls) it is necessary to define precisely LOS with respect to displacement of COM. The examination of the above mentioned issue requires application of sophisticated research tools of optoelectronic systems for kinematics type or force platforms enabling the indirect estimation of displacements of COM (e.g. Neurocom Pro-Balance Master). However, defining a less precise (from the perspective of the definition) but reliable coefficient, which would be at the same time simple in implementation and interpretation, may offer a sufficient solution. The method seems to meet the above mentioned criteria whereas earlier research showed that the evaluation of LOS_COP_ may be useful to evaluate the risk of falls. The analysis of LOS_COP_ results depicted that non-falling men represented higher values than falling men (Stemplewski et al., 2003). However, further research shall be conducted in that aspect, especially a follow-up study.

A similar study was conducted by Juras et al. (2008), who evaluated seven different components of the COP signal during a maximal voluntary forward lean in three stages of the test (quiet stance, leaning and maintenance of leaning position). The highest reliability was reported in the case of components characterizing the range of COP stated in absolute values and between the mean COP position in the phase of a quiet stance and the phase of maintenance of leaning position which is consistent with own study. There are also reports concerning the evaluation of the reliability of the measurement with the application of Neurocom Pro-balance Master platform, where LOS were analyzed as the percentage of the angular deflection of COM from the perpendicular with respect to theoretical limits of stability (6.25º forward, 4.45º backward and 8º leftward and rightward). In general, low values of ICC were reported for individual parameters (Brouwer et al., 1998 – examination of a group of subjects in the age of 20–32 years; Dodd et al., 2003

– examination of elderly persons with surgery of hip fractures).

The results of the study showed very high internal consistency and stability of results for COP parameters obtained during maximal voluntary body leaning. This kind of evaluation may be useful in assessment of actual functional status as well as in assessment of changes in this status among elderly people.

## Conclusions

1. In case of COP displacement measures in quiet standing, for most parameters related to the spatial distribution of COP, rather low coefficients of reliability were reported which showed a low application value of those parameters in case of evaluation of the risk of falls among elderly people. The only parameter that showed a high internal consistency and stability in time was the COP average velocity.
2. Parameters related to the evaluation of stability limits (LOS_COP_) showed a very high internal consistency and stability of results. The method is simple in the application and does not require the implementation of advanced research tools which may constitute its significant advantage from the perspective of its potential implementation into clinical practice.

## Perespectives

The reliability of parameters of COP displacement during a quiet stance and during voluntary body leaning in AP and ML directions were evaluated among elderly men in this study. In case of quiet stance measurements, probably further work should be focused on reliability studies of differences between signals of COP and COM which gives more precise information about postural control. In the case of evaluation of approximated limits of stability, there is a need for more studies regarding application of these measurements for evaluation of fall risk among elderly (especially follow-up studies). Further studies including people with different disabilities may also indicate a possible role in evaluation of progress in therapy.

## Figures and Tables

**Table 1:** Values of the intraclass correlation coefficients, confidence intervals, and number of tests which had to be averaged to achieve intraclass correlation coefficient equal or higher than .90 for COP displacement measures in quiet standing obtained during an intrasession and intersession retest

	ICC_2,1_	95%CI	ICC_2,k_	95%CI	*k* ICC≥ .90
*Intrasession (4 trials - the same day, 2 min. break between trials)*					
Y_Range_	.42	.23 – .63	.74	.54 – .87	13
X_Range_	.51	.32 – .70	.81	.66 – .90	9
MR	.46	.26 – .66	.77	.59 – .88	11
V_Avg_	.84	.74 – .92	.96	.92 – .98	2
Area	.61	.43 – .77	.86	.75 – .93	6
*Intersession (4 trials one week apart)*					
V_Avg_	.76	.62 – .87			

ICC_2,1_ – intraclass correlation coefficient for single ratings; ICC_2,k_ – intraclass correlation coefficient for averages of k ratings; CI – confidence intervals; k ICC≥ .90 – number of tests which had to be averaged to achieve ICC≥ .90 X and Y_Range_ – distance between maximal and minimal position of COP in frontal and sagittal planes, respectively; MR – average radial position vector length (mean radius); V_Avg_ – average velocity; Area – sway area limited with an ellipse of the 95^th^ percentile

**Table 2:** Values of the intraclass correlation coefficients, confidence intervals, and number of tests which had to be averaged to achieve intraclass correlation coefficient equal or higher than .90 for stability limits measurement obtained during an intersession and intersession retest

	ICC_2,1_	95%CI	ICC_2,k_	95%CI	*k* ICC≥ .90
*Intrasession (4 trials - the same day, 2 min. break between trials)*					
F_max_	.87	.79 – .93	.97	.94 – .98	2
B_max_	.82	.71 – .90	.95	.91 – .97	2
FB	.92	.87 – .96	.98	.96 – .99	1
R_max_	.94	.90 – .97	.99	.97 – .99	1
L_max_	.95	.92 – .97	.99	.98 – .99	1
RL	.96	.93 – .98	.99	.98 – .99	1
LOS_COP_	.96	.92 – .98	.99	.98 – .99	1
*Intersession (4 trials one week apart)*					
F_max_	.79	.66 – .88			
B_max_	.67	.51 – .81			
FB	.78	.65 – .88			
R_max_	.84	.73 – .91			
L_max_	.82	.71 – .90			
RL	.85	.76 – .92			
LOS_COP_	.85	.75 – .92			

ICC_2,1_ – intraclass correlation coefficient for single ratings; ICC_2,k_ – intraclass correlation coefficient for averages of k ratings; CI – confidence intervals; k ICC≥ .90 – number of tests which had to be averaged to achieve ICC≥ .90F_max_, B_max_ - maximal (forward) and minimal (backward) displacement from the centroid data in sagittal plane; R_max_, L_max_ – maximal (rightward) and minimal (leftward) displacement from the centroid data in frontal plane; FB and RL – distance between maximal and minimal position of COP in sagittal and frontal planes, respectively; LOS_COP_ – calculated area of limits of stability
